# Supplementary material for: A multiscale model of immune surveillance in micrometastases gives insights on cancer patient digital twins
Source: NPJ Syst Biol Appl. 2024 Dec 4;10:144. doi: 10.1038/s41540-024-00472-z (PMC11614875; doi:10.1038/s41540-024-00472-z)
Supplement: Supplementary file 1 — Supplementary information [file 41540_2024_472_MOESM1_ESM.pdf]

Supplementary Information to: A multiscale model of immune surveillance in micrometastases gives insights on cancer patient digital twins

Heber L. Rocha[1], Boris Aguilar[2], Michael Getz[1], Ilya Shmulevich[2†] and Paul Macklin[1*]

[1]Intelligent Systems Engineering, Indiana University, Bloomington, IN, USA.
[2]Institute for Systems Biology, Seattle, WA, USA.

*Corresponding author(s). E-mail(s): macklinp@iu.edu;
†*In memoriam*

Supplementary Note 1: Parameters tables

The multiscale model that has been constructed contains hundreds of parameters. In Tables 1 to 3, we present the essential parameters defined for a hypothetical middle patient, which represents the average characteristics of all patients. The set of virtual patients was created based on ten parameters that are associated with the immune response. Out of these ten parameters, six are linked to the recruitment of dendritic cells and macrophages into the microenvironment ($r_{recruit}[MP]$, $\rho_{min}[MP]$, $\rho_{sat}[MP]$, $r_{recruit}[DC]$, $\rho_{min}[DC]$, and $\rho_{sat}[DC]$), three are associated with the production of T cells in the lymph node (δ_C, κ_T, and δ_{DM}), and one is related to the migration of activated dendritic cells from the microenvironment to the lymph node (r_{leave}). The parameter range values for generating the virtual patient dataset using Latin Hypercube Sampling (LHS) are shown in Table 4.

Supplementary Table 1: Table of motility, substrate secretion, and recruitment parameters.

Parameter	Description	Value	Unit	Reference
σ_{TNF}	sensitivity of chemotaxis along with TNF gradient	10	–	Estimated
σ_{debris}	sensitivity of chemotaxis along with debris gradient	1	–	Estimated
s_{mot}[DC, CD4+]	speed motility of DCs and CD4+ T cells	2	$\mu m/min$	[1]
s_{mot}[M0, CD8+]	speed motility of M0, and CD8+ T cells	4	$\mu m/min$	[1]
s_{mot}[M1,M2]	speed motility of M1 and M2	0.4	$\mu m/min$	[1]
T_{per}	persistence time on velocity direction	5	min	[1]
b[MP, DC, CD4+]	migration bias of DCs, MPs, and CD4+ T cells	0.7	–	[1]
b[CD8+]	migration bias of CD8+ T cells	0.3	–	[1]
U_{TNF}[DC,M0,CD8+]	TNF uptake rate of DCs, M0, and CD8+ T cells	0.01	min^{-1}	Estimated
U_{TNF}[M1,M2]	TNF uptake rate of M0 and M1	0	min^{-1}	Estimated
U_{debris}[DC,M0,CD8+]	debris uptake rate of DCs, M0, and CD8+ T cells	0.1	min^{-1}	[1]
S_{TNF}[M1,M2]	TNF secretion rate of M0 and M1	1	min^{-1}	Estimated
S_{debris}[Dead cells]	debris secretion rate of all dead cells	1	min^{-1}	[1]
$r_{recruit}$[MP]	recruitment rate of macrophages	4.0×10^{-9}	$cell/min \cdot \mu m^3$	[1]
$r_{recruit}$[DC]	recruitment rate of dendritic cells	2.0×10^{-9}	$cell/min \cdot \mu m^3$	[1]
ρ_{min}[MP]	minimal signal of TNF to recruit macrophages	0.1	TNF concentration	[1]
ρ_{min}[DC]	minimal signal of TNF to recruit DCs	0.1	TNF concentration	[1]
ρ_{sat}[MP]	saturation signal of TNF to recruit macrophages	0.3	TNF concentration	[1]
ρ_{sat}[DC]	saturation signal of TNF to recruit DCs	0.3	TNF concentration	[1]

Supplementary Table 2: Table of phagocytosis, attachment, mechanical parameters.

Parameter	Description	Value	Unit	Reference
r_{phag}	phagocytosis rate of macrophages	0.167	min^{-1}	[1]
V_{max}	Max volume of macrophages to stop cell engulfment	6.5×10^3	μm^3	[1]
r_{death}	death rate of exhausted macrophages	0.01	min^{-1}	[1]
$r_{V_{int}}$	intracellular digestion rate in macrophages	1	$\mu m^3/min$	[1]
r_{attach}[DC]	attachment rate of dendritic cells	100.0	min^{-1}	Estimated
r_{leave}	DC exit rate from the tumor microenvironment	0.2	min^{-1}	Estimated
$\bar{r}_{01}$[CD8+ nearby DC]	DC-induced CD8+ T cell cycle entry rate	2.08×10^{-3}	min^{-1}	[1]
r_{attach}[CD8+ nearby DC]	attachment rate of CD8+ induced by DC	0.6	min^{-1}	[1]
r_{attach}[CD8+]	attachment rate of CD8+	0.2	min^{-1}	[1]
T_{attach}	the mean duration of CD8+ and cancer cells attachment	8.5	min	[1]
r_E	coefficient for the magnitude of the elastic force	5.0×10^{-2}	min^{-1}	[2]
r_P	relaxation rate according a plastic reorganization	5.0×10^{-4}	min^{-1}	[2]
d_P	maximum deformation of parenchymal cells	0.75	μm	[2]
$\bar{r}_{01}$	base rate for entry of cancer cells into the cell cycle	2.0×10^{-3}	min^{-1}	Estimated
p_{max}	maximum simple pressure to cancer cells proliferate	10	–	Estimated

Supplementary Table 3: Table of parameters associated with lymph node and tumor microenvironment interaction.

Parameter	Description	Value	Unit	Reference
τ_{DC}	time to DCs migrate to lymph node	720	min	[1]
k_D	antigen presentation rate	1	min^{-1}	[1]
δ_{DM}	natural decay rate of D_M	3.5×10^{-4}	min^{-1}	[1]
$\sigma_{T_{H1}}$	proliferation rate of T_{H1}	7.0×10^{-4}	$cells^2/min$	[1]
$\pi_{T_{H1}}$	activation rate of T_{H1}	1.5×10^{-5}	min^{-1}	[1]
$\delta_{T_{H1}}$	deactivation rate of T_{H1}	7.0×10^{-7}	$cell^{-1}min^{-1}$	[1]
β	half maximum deactivation of T_{H1}	5.0×10^{2}	$cells$	[1]
μ_{T_H}	natural death rate of T_{H1} and T_{H2}	1.56×10^{-5}	min^{-1}	[1]
$\sigma_{T_{H2}}$	proliferation rate of T_{H2}	2.8×10^{-5}	$cells/min$	[1]
$\pi_{T_{H2}}$	activation rate of T_{H2}	2.0×10^{-6}	$cell^{-1}min^{-1}$	[1]
ρ	weight conversion between T_{H1} and T_{H2} activation	1	$cell$	[1]
π_T	activation rate of T cells	3.0×10^{-3}	min^{-1}	[1]
ϕ_T	scaling factor between lymph node and tumor microenvironment	1.0×10^{6}	$cells$	[1]
β_{T1}	half maximum of activation of T cells	2.0×10^{3}	$cells$	[1]
δ_T	deactivation rate of T cells	7.0×10^{-4}	min^{-1}	[1]
β_{T_2}	half maximum deactivation of T cells	2.0×10^{2}	$cells$	[1]
δ_C	natural decay rate of T cells	1.4×10^{-6}	min^{-1}	[1]
ϕ_C	population threshold of T cells	10	cells	[1]
κ_T	recruitment rate of T_{Ct} and T_{ht}	1.1×10^{-4}	min^{-1}	[1]
τ_{TC}	time to T_{Ct} and T_{ht} infiltrate the tumor microenvironment	360	min	[1]

Supplementary Table 4: Range of parameters of virtual patients.

Parameter	Description	[Min, Max]	Unit
$r_{recruit}[MP]$	recruitment rate of MP	$[0, 8.0 \times 10^{-9}]$	$cell/min \cdot \mu m^3$
$\rho_{min}[MP]$	minimal signal of TNF to recruit MP	$[0, 0.2]$	TNF conc.
$\rho_{sat}[MP]$	saturation signal of TNF to recruit MP	$[0, 0.6]$	TNF conc.
$r_{recruit}[DC]$	recruitment rate of DCs	$[0, 4.0 \times 10^{-9}]$	$cell/min \cdot \mu m^3$
$\rho_{min}[DC]$	minimal signal of TNF to recruit DCs	$[0, 0.2]$	TNF conc.
$\rho_{sat}[DC]$	saturation signal of TNF to recruit DCs	$[0, 0.6]$	TNF conc.
r_{leave}	DC exit rate from the TME	$[0, 0.4$	min^{-1}
δ_C	natural decay rate of T cells	$[0, 2.8 \times 10^{-6}]$	min^{-1}
κ_T	recruitment rate of T_{Ct} and T_{ht}	$[0, 2.2 \times 10^{-4}]$	min^{-1}
δ_{DM}	natural decay rate of D_M	$[0, 7.0 \times 10^{-4}]$	min^{-1}

Subject to $\rho_{min}[DC] < \rho_{sat}[DC]$ and $\rho_{min}[MP] < \rho_{sat}[MP]$ constraints.

Supplementary Note 2: Data compression

The multiscale model generates high-dimensional data with spatial and temporal information. According to our specifications, each simulation stores 31 snapshot images over ten days of evolution, resulting in a total dataset size of approximately 5.55 TB. To simplify and reduce the dataset size, we perform a massive dimensionality reduction on the snapshot images, condensing the entire dataset to 6 GB. The arrangement of cells and substrates in the microenvironment at a specific simulated snapshot is encapsulated in a 24-bit 5x5 RGB bitmap image, where each channel encodes specific information:

- **Red channel (first 8 bits):** Stores information on the presence or absence of parenchymal cells, cancer cells, and dead immune cells.
- **Green channel (next 8 bits):** Represents the presence of live immune cells.
- **Blue channel (last 8 bits):** Specifies concentration ranges of TNF (tumor necrosis factor) and debris (as defined in Table 5).

For example, consider a pixel where there are live cancer cells, macrophage M0, and voxels with debris concentrations in the range of [0.2, 0.3], with a TNF concentration 0. According to Table 5, the pixel color in RGB will be:

$$\begin{bmatrix} 0 & 0 & 0 & 0 & 0 & 0 & 0 & 1 \\ 0 & 1 & 0 & 0 & 0 & 0 & 0 & 0 \\ 1 & 1 & 0 & 0 & 1 & 0 & 0 & 0 \end{bmatrix} \begin{bmatrix} 2^0 \\ 2^1 \\ \vdots \\ 2^7 \end{bmatrix} = \begin{bmatrix} 128 \\ 2 \\ 19 \end{bmatrix}$$

This approach allows us to drastically reduce the output space of the model, admitting it comes at the cost of losing some data resolution while retaining crucial aspects of the spatial and temporal dynamics.

The conversion process is accomplished by mapping the computational domain into partitions that define the pixels of the bitmap image. Within

each pixel, we calculate the presence (bit 1) or absence (bit 0) of specific cell phenotypes and concentrations of relevant chemicals, such as TNF and debris. Figure 1 illustrates the data compression for three different immune control scenarios of micrometastases on day ten (NC, MC, and SC).

Supplementary Table 5: Signature of the bits in RGB image during the process of data compression.

Bit position	Red Channel	Green Channel	Blue Channel
1	live parechymal cell	inactivated DC	$0.00 \leq \rho_{debris} < 0.25$
2	dead parechymal cell	macrophage M0	$0.25 \leq \rho_{debris} < 0.50$
3	dead cacner cell	exhausted MP	$0.50 \leq \rho_{debris} < 0.75$
4	dead DC	hyperactivated MP	$\rho_{debris} \geq 0.75$
5	dead MP	live CD4+ T cell	$0.00 \leq \rho_{TNF} < 0.25$
6	dead CD4+ T cell	activated DC	$0.25 \leq \rho_{TNF} < 0.50$
7	dead CD8+ T cell	activated MP	$0.50 \leq \rho_{TNF} < 0.75$
8	live cancer cell	live CD8+ T cell	$\rho_{TNF} \geq 0.75$

DC: dendritic cell, M0: inactivated macrophage, MP: macrophage, ρ_{TNF}: concentration of tumor necrosis factor, ρ_{debris}: concentration of debris.

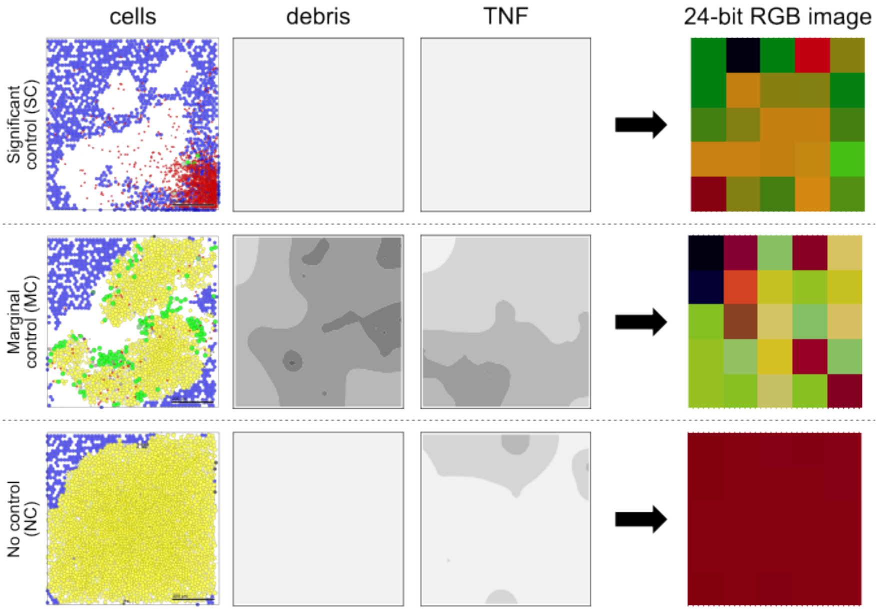

Supplementary Figure 1: The spatial data compression into a 24-bit RGB image from the multiscale model of immune surveillance in micrometastases.

Supplementary Note 3: Analysis of clustering of all trajectories

We conducted an investigation comparing two distinct clusters of NC-labeled trajectories resulting from dimensionality reduction using Uniform Manifold Approximation and Projection (UMAP) across a dataset of 100,000 virtual patient trajectories. Each trajectory is represented by 31 snapshots of 5x5 bitmap RGB images. Our findings reveal significant differences in immune system activation between these clusters. Cluster 1, located on the left side in Figure 2, exhibits a heightened level of immune system activation. In contrast, Cluster 2 on the right side in Figure 2 displays a decline in immune system activity. Our comprehensive analysis underscores differences in the composition of immune cell populations within these two samples. Upon obtaining additional samples, we discerned that the primary dissimilarity lies in the production of T cells within the lymph nodes. Cluster 1 consistently exhibits an upward trajectory in T cell production, while Cluster 2 reaches a state of saturation. T cell production relies entirely on immune activation, specifically driven by the activation and migration of dendritic cells (DCs). These DCs, in turn, trigger T cell production.

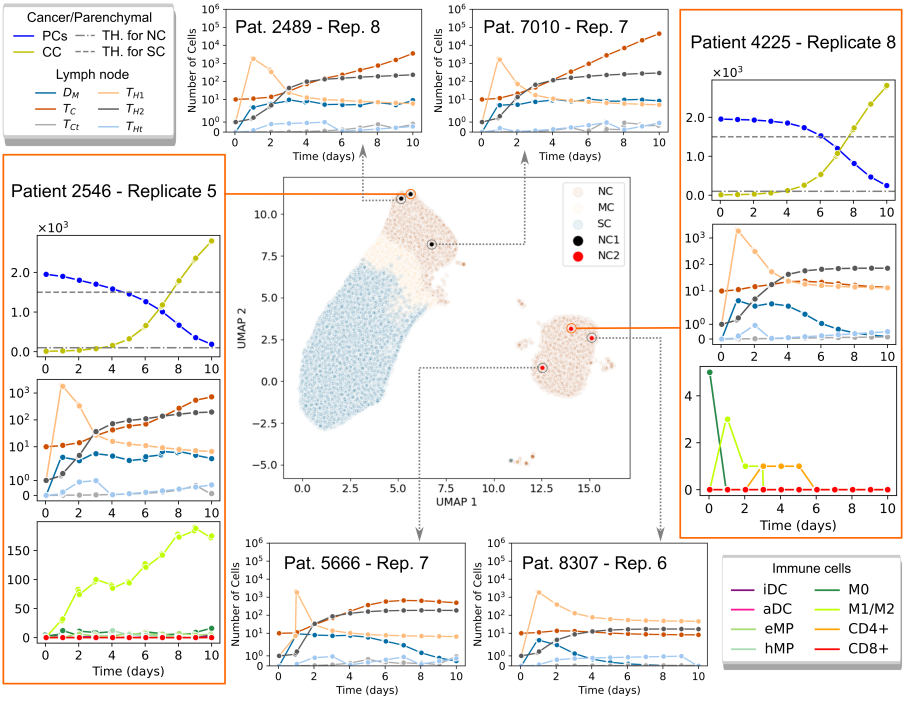

Supplementary Figure 2: Visualization of Spatial and Temporal Trajectories in the No Control (NC) Case. In the reduced space represented by Uniform Manifold Approximation and Projection (UMAP), this figure showcases the spatial/temporal trajectories of all patients in the no control (NC) case. The samples denoted by orange arrows correspond to cell population plots of cancer cells, parenchymal cells, lymph node (LN) cells, and immune cells within the microenvironment. Meanwhile, the samples marked with gray arrows emphasize distinctions between clusters. Notably, the cluster on the right in the NC case exhibits a higher population of T cells within the lymph node compared to the left cluster, indicative of immune system activation.

Supplementary Note 4: Classification analysis

We perform time series clustering with the Python library tslearn [3] with k-means and the dedicated time series metric, Dynamic Time Warping (DTW). This metric effectively addresses the issue of time shifts dependencies in the trajectories. In the confusion matrix (Table 6), we compare this clustering with the classifier strategy adopted in this work, based on a cancer population thresholds on day 10 of the simulation used in this manuscript. Notably, both classifications exhibit considerable similarities, with Cluster 0 and 1 capturing our extreme case labels significant control (SC) and no control (NC), respectively, with over 90% accuracy. Figure 3 corroborates this finding, displaying the average and standard deviation for each group along with the

100,000 trajectories. Altogether, this analysis suggests that our classifier have low dependence on individual time variations but relies on the entire trajectory dynamics.

Supplementary Table 6: Normalized confusion matrix for the classification based on the threshold of the cancer cell population on day 10, along with the time series clustering over a 10-day period using DTW.

	Sign. Control (SC)	No Control (NC)	Marg. Control (MC)	Total*
Cluster 0	0.92	0.00	0.08	41,035
Cluster 1	0.00	1.00	0.00	44,775
Cluster 2	0.27	0.03	0.70	14,190
Total*	41,808	45,149	13,043	100,000

* Total number of trajectories.

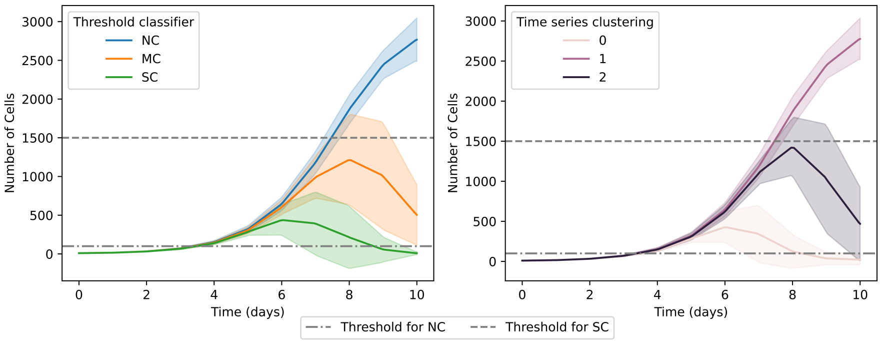

Supplementary Figure 3: The comparison of live cancer cell population over time. Data obtained from 100,000 trajectories using our patient classifier and dynamic time warping.

Supplementary Table 7: Trajectories of four patients displaying different immune response classifications. Patient 6 shows multiple labels across trajectories (NC, SC, MC), patient 2 shows all trajectories classified as no control (NC), patient 93 shows all trajectories classified as marginal control (MC), and patient 5 shows all trajectories classified as significant control (SC). Each row is organized by the specific label.

ID	Label	Cancer/Parenchymal	Lymph Node	Immune
6	NC	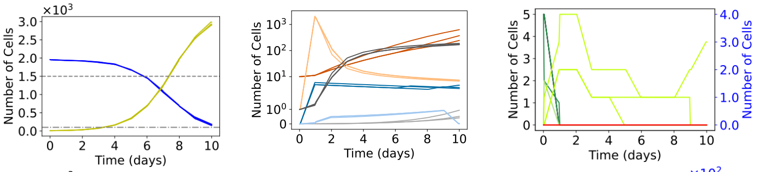		
6	MC	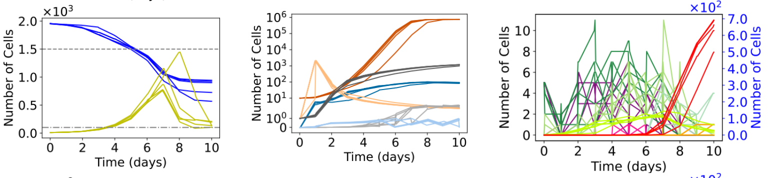		
6	SC	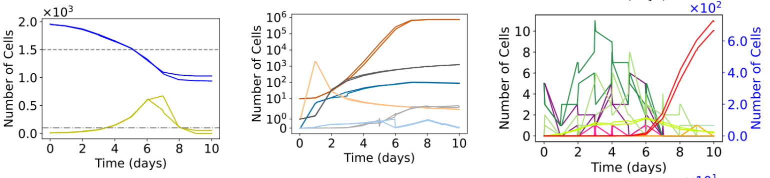		
2	NC	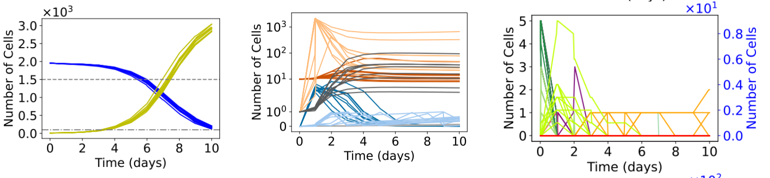		
93	MC	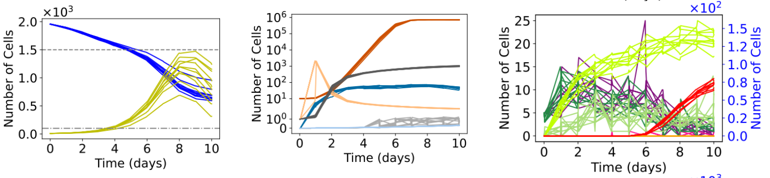		
5	SC	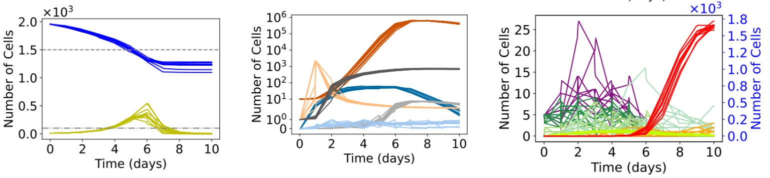		

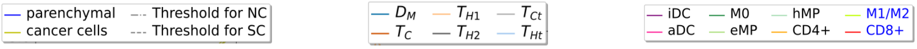

$\mathbf{D_M}$: DCs that have migrated from the TME to the LN, $\mathbf{T_{H1}}$: helper T cells type one, , $\mathbf{T_{H2}}$: helper T cells type two, $\mathbf{T_C}$: cytotoxic T cells, $\mathbf{T_{Ct}}$: CD8+ cytotoxic T cells, $\mathbf{T_{ht}}$: CD4+ helper T cells, **iDC**: inactive DC, **aDC**: active DC, **eMP**: exhausted macrophages, and **hMP**: hyperactivated macrophages.

Supplementary References

[1] Getz, M., Wang, Y., An, G., Asthana, M., Becker, A., et al.: Iterative community-driven development of a SARS-CoV-2 tissue simulator. Preprint at https://www.biorxiv.org/content/10.1101/2020.04.02.019075v4 (2021)

[2] Wang, Y., Brodin, E., Nishii, K., Frieboes, H.B., Mumenthaler, S.M., *et al.*: Impact of tumor-parenchyma biomechanics on liver metastatic progression: a multi-model approach. Scientific reports **11**(1), 1–20 (2021)

[3] Tavenard, R., Faouzi, J., Vandewiele, G., Divo, F., Androz, G., Holtz, C., Payne, M., Yurchak, R., Rußwurm, M., Kolar, K., Woods, E.: Tslearn, a machine learning toolkit for time series data. Journal of Machine Learning Research **21**(118), 1–6 (2020)
